# Editorial

**Published:** 2013-09-25

**Authors:** VL Purcarea

**Affiliations:** Carol Davila University of Medicine and PharmacyRomania

“Carol Davila” University of Medicine and Pharmacy has organized for the first time a national congress with an international participation, with the theme “INITIATION, EVOLUTION, EXCELENCY”, in the imposing building of the House of Parliament of Romania, during the 30th of May – 1st of June 2013.

**Photo 1:**
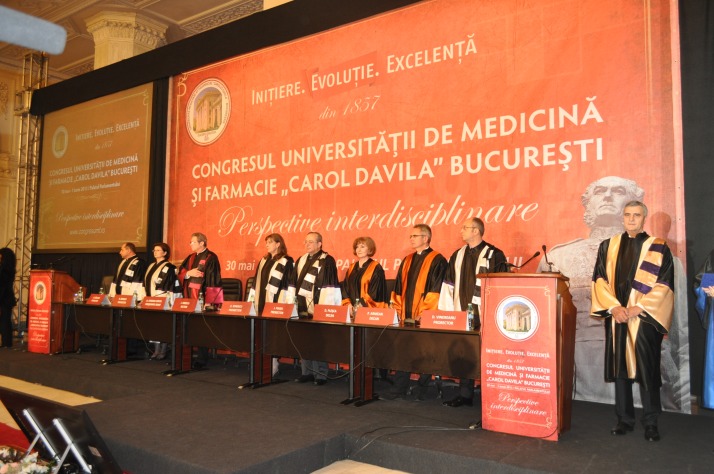
“Carol Davila” University of Medicine and Pharmacy Leadership

In 156 years of existence, the prestigious school has given the country and the world some very valuable personalities, among whom we should mention Carol Davila, Nicolae Kretzulescu, Victor Babes, Thoma Ionescu, George Marinescu, Ion Cantacuzino, Francisc Rainer, George Emil Palade.

“Carol Davila” University of Medicine and Pharmacy has gained a well deserved prestige in the elite of the Romanian medicine and pharmacy schools thanks to the existence of a high academic, didactic and scientific teaching staff, the excellent professional level of the generations of graduates, recognized both in Romania and abroad, the performances in Romania, the international performances and also the impressive editorial and publishing activity. From the 9400 students, over 1400 are foreigners and over 1300 are PhDs, benefiting from the guidance and experience of over 1600 professors. The General Mayor of Bucharest, Prof. Dr. Sorin Oprescu and the Ministry of National Education, Prof. Dr. Remus Pricopie have honored with their presence in the Congress. 

**Photo 2:**
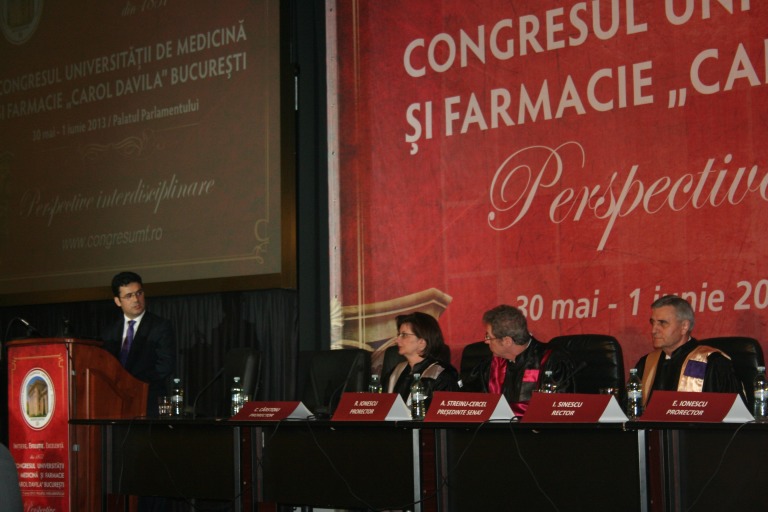
Ministry of National Education, Prof. Dr. Remus Pricopie leading the discussion

**Photo 3:**
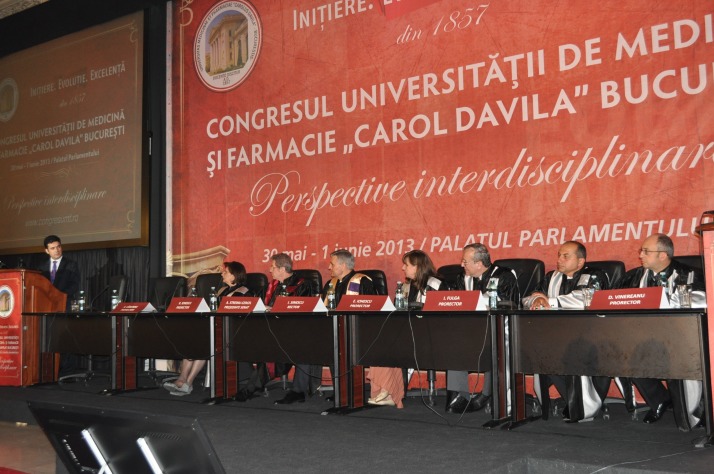
Ministry of National Education, Prof. Dr. Remus Pricopie and “Carol Davila” University Leadership

Very interesting subjects have been debated in the congress: “From the cells of the cardiovascular system to cardiovascular prevention”; “Urology and nephrology – a perfect relationship”; “Pediatric medicine: challenges of the year 2013”; “Arterial pulmonary hypertension and the systemic diseases: what is new?”;” New challenges in oncology”; “From the molecular investigations to the therapeutic challenges in current clinical practice”; “Cardio-neurology: what is new?”; “Infectious diseases with multidisciplinary implications”; “Dentistry and systemic diseases”, “Pharmacology in current clinical practice”, “Obesity – agony and ecstasy”; “Pregnancy and the systemic diseases”, papers which have generated an alive interest for the specialists. The congress has also included some satellite symposiums and an extremely useful session of posters presentation. 

Moreover, on the occasion of the congress, two Doctor Honoris Causa ceremonies have taken place and 6 Doctor Honoris Causa titles have been awarded. 

On the 30th of May, Prof. Antonio Federico, Prof. Martin Burian and Prof. Piero Portincasa have been awarded the honorable title Doctor Honoris Causa. 

**Photo 4:**
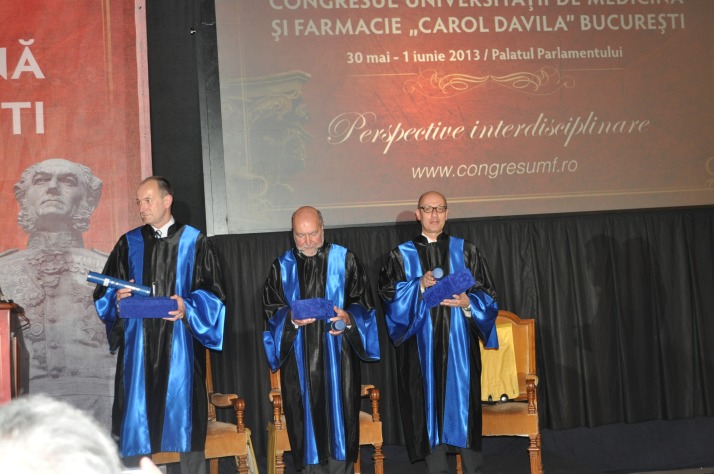
Prof. Martin Burian, Prof. Antonio Federico and Prof. Piero Portincasa

Prof. ANTONIO FEDERICO, a valuable and prominent personality in the field of Neurology and Neurosciences, the author of over 500 scientific articles and of numerous treaties and chapters in important neurology treaties, published in some of the most important international medical publishing houses. His articles have 6000 citations in journals which are indexed in Thomson ISI, his Hirsch Index being 36. Prof. Antonio Federico is the Editor-in-chief of the prestigious publication “Neurological Sciences”. 

Prof. MARTIN BURIAN is the Chief of Otorhinolaryngology and Surgery of Head and Neck Department in Barmherzingen Schwestern Linz Hospital, Austria, has done 3400 surgeries/ year and the medical assistance of over 20000 patients/ year in ambulatory regimen, is a wonderful surgeon with an inexhaustible thirst for knowledge, but, he is also a great professor. The scientific, didactic and surgical preoccupations of Prof. Martin Burian are relevant in the field of head and neck oncological surgery, as well as reported to the endoscopic and Laser surgical approach of the nasal pathology, the functional and cosmetic surgery in the otorhinolaryngology field. The publishing and editorial activity bears the mark of the fundamental preoccupation for the oncological surgery of the head and neck, for the tumors localized in the ORL field, as well as for the complex chemotherapy and radiotherapy treatment of this pathology, including the publishing of some original personal studies regarding the innervation and the electromyography of the trapeze muscle, regarding the anatomy of the lachrymal canal, the electroneurophysiology of the face muscle, the physiopathology of the salivary glands, as well as the preoccupations regarding the gene expression in squamous-cellular carcinomas of the head and neck or some new chemotherapy protocols, diagnostic (photodynamic diagnosis) protocols or a treatment (electrophoresis) associated with the primary malign or recurrent tumors of the head and neck.

Prof. PIERO PORTINCASA, professor of Internal Medicine in Bologna University, professor of Internal Medicine in the Medical School of Bari, President of the Internal Medicine Residency Program, Member of the Academy of Science in Apulia, responsible member of the ERASMUS Program for the University of Medicine in Bari, President of the European Society for Clinical Investigations, Co-editor of the European Journal for Clinical Investigations (Wiley-Blackwell), Member of Honor of the Romanian Society of Gastroenterology and Hepatology, Chief of the Diagnostic and Semiology Unit in “A Murri” Medical Clinic, University of Bari, vice-director of “A Murri” Medical Clinic, University of Bari. Professor Piero Portincasa is involved in the European mobility programs formation (Socrates-Erasmus Lifelong Learning) and is also the leader of a famous international research group in Bari, being involved in translational research, focusing on some aspects of the hepatobiliary diseases (liver, cholecyst, intestine) and the physiopathology gastro-intestinal secretions, transport and motility. 

The ceremony of awarding the honorable title for Prof. Christopher Bull Granger, Prof. Mark Jean Cristophe Humbert and Prof. Frank M. Rümmele has taken place on the 31st of May 2013. 

**Photo 5:**
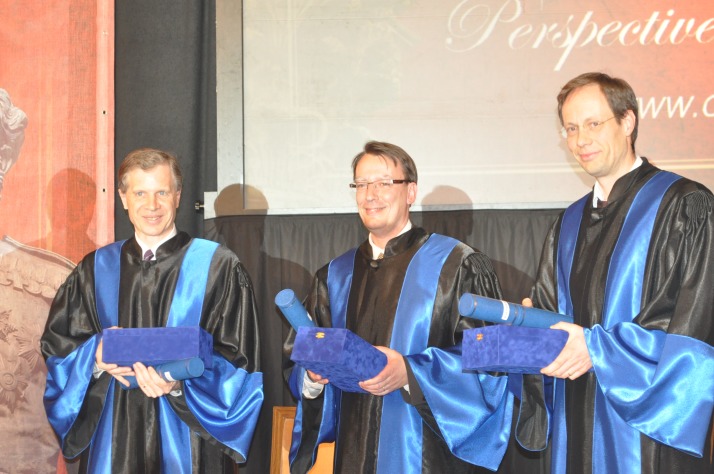
Prof. Martin Burian, Prof. Antonio Federico and Prof. Piero Portincasa

Prof. CHRISTOPHER BULL GRANGER, from Duke University of Medicine, one of the most famous universities in the world, is currently director of Cardiac Care Unit in the cardiology division. Much of his activity has been dedicated to clinical research. The publishing activity of Prof. Granger is sensational: 562 articles published in international journals, most of them in New England Journal of Medicine, The Lancet, JAMA, Circulation and European Heart Journal. Among them, 426 are original articles, very important for the cardiologic research and 136 are reviews and editorials. Moreover, Prof. Granger has published 24 books and book chapters in important international treaties such as the ones edited by Rob Califf, Eric Topol, or Eugene Braunwald. Prof. Christopher Granger is a member of the editorial committees of 8 international cardiology journals. 

He is a fellow of the American College of Cardiology, American Heart Association and European Society of Cardiology. For his research and publishing activity he has received Top 10 prize twice, for research in USA, in 2007 and 2012. He had a decisive contribution in developing a research institute in Brazil, being a member of honor of the Brazilian Society of Internal Medicine. 

Prof. FRANK M. RÜMMELE, professor of pediatrics in Rene Descartes University of Medicine in Paris, responsible with the intestinal immunopathology program in the pediatrics department and is the Chief of the Pediatric Gastroenterology Clinic in Necker Enfants Malades Hospital. Prof. Frank Rümmele is currently the P-ECCO chief, GETAID president – the pediatric branch, vice-president of the Francophone Group of Gastroenterology, Hepatology and Nutrition (GFHGNP), vice-president of ESPGHAN working group on intestinal inflammatory diseases. He has gained lots of titles and accreditations, already having over 15 prizes and distinctions received for different scientific researchers presented and/ or published, the most recent distinction being received in 2011: Laureate of Sorbona University in Paris with the excellency prize for scientific research. He is the coordinator of numerous research grants and the author of over 100 original papers published in very prestigious journals and he is the author of many prestigious books in pediatrics gastroenterology (Inflammatory Bowel Diseases, Pediatric Gastrointestinal diseases, etc.). His preoccupations have been oriented towards the aspects concerning the anatomical and immunological modifications in the intestinal inflammatory affections which appear in pediatric pathology. 

Prof. MARC HUMBERT, is the president of South Paris Research Center for Care in Pulmonary Hypertension, Director of INSERM Unit 999 “Pulmonary hypertension: pathophysiological and innovative therapies”. His international consecration has brought him the title of President of the Social European Group for Respiration “Pulmonary circulation and vascular diseases”. His scientific papers and research have offered him the possibility to publish papers in fields such as asthma, pulmonary tension and pulmonary inflammation. These accomplishments have brought the author over 290 reviews of the articles in the most famous journals in the world (New England Journal of Medicine, Lancet, Nature, Nature Med, J Clin Invest, J Exp Med), as well as in top journals in the field of problems with breathing and cardiovascular medicine (Am J Respir Crit Care Med, J Am Coll Cardiol, Circulation, Circ Res, etc.). He has accumulated 13000 citations with 40 papers cited over 100 times, two of them being cited for more than 600 times. His Hirsch Index is 61. In 2006, he has been awarded the title European Respiratory Society Cournand Lecture (Course on the theme “The burden of pulmonary hypertension”), a special distinction for a young scientific personality. In 2009, the Royal Academy of Arts and Sciences in Netherlands has offered him the Descartes-Huygens Prize. 

In 2010 he was awarded the title of vice-president of Assistance Publique Hopitaux de Paris, responsible for the research department and, currently, he is the president of AP-HP Research Committee. 

The members of the Students Choir of “Carol Davila” University of Medicine and Pharmacy have brought their important contribution in intensifying these emotional ceremonies.

**Photo 6:**
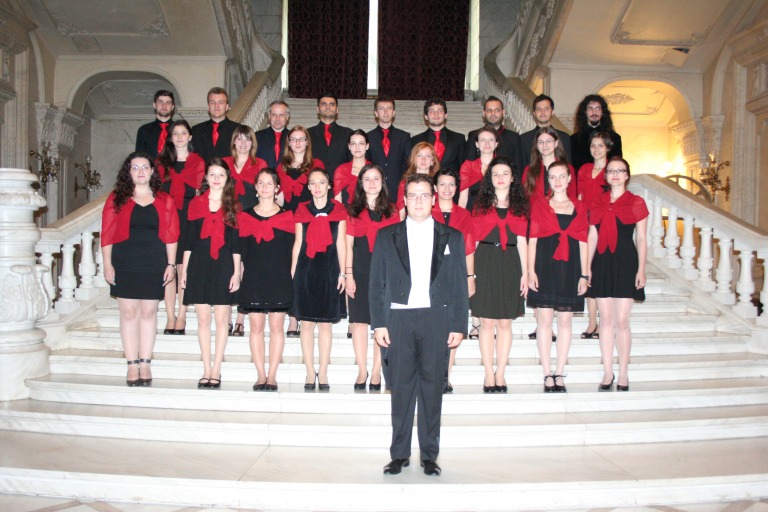
Students Choir of “Carol Davila” University of Medicine and Pharmacy

The congress had an exceptional scientific value and the presence of over 1700 participants is an alive proof of the success of the event. The generous efforts of the University together with the rigorous and efficient effort of the organizers have given value and academic performance to the congress. 

Purcarea VL, MD, PhD, Eng

Executive Editor

